# PREVALENCE AND DISPARITIES OF VERY LOW-INCOME OLDER ADULT RENTERS IN US METROPOLITAN AREAS

**DOI:** 10.1093/geroni/igad104.1652

**Published:** 2023-12-21

**Authors:** Anthony Traver, Holly Dabelko-Schoeny

**Affiliations:** The Ohio State University, Columbus, Ohio, United States; The Ohio State University, Columbus, Ohio, United States

## Abstract

Income eligibility for federal housing subsidies is based on HUD calculations of area median income (AMI) and family size. Frequently used measures of poverty and income do not reflect the geographic economic variations on which HUD eligibility is based. By integrating Current Population Survey (CPS) data for metropolitan-dwelling people in 2000, 2010, and 2020 with HUD yearly 50% AMI thresholds (very low-income (VLI)), this study aims to provide a novel estimate of the number of metropolitan older adult (OA) (62+) VLI renters and to compare their growth rate, rental assistance use, race, and health with other population groups. Sample weights are used to produce national estimates of four metropolitan OA subgroups: VLI renters, VLI owners, non-VLI renters, and non-VLI owners. Results show that between 2000 and 2020, the total number of metropolitan OAs increased by an estimated 84%, OA VLI renters increased by an estimated 74%, and OA VLI renters without rental assistance increased by an estimated 71%. Across all years, metropolitan VLI OA renters were more likely to be non-white, disabled, report poorer health, and receive rental and utility assistance. Approximately two-thirds of metropolitan VLI OA renters did not receive any form of rental assistance across time points. VLI OA renters without rental assistance face significant affordability challenges as metropolitan rental prices rise faster than most retirement incomes. Communities should proactively link their growing OA VLI renter populations with financial assistance and supportive services to reduce their risk of housing precarity in late life.

